# Four‐dimensional computed tomography can visualise the in vivo glenoid track and quantify dynamic glenohumeral contact mechanics during active motion in subjects with shoulder instability: A preliminary study

**DOI:** 10.1002/jeo2.70807

**Published:** 2026-06-16

**Authors:** Keigo Honoki, Kyosuke Numaguchi, Daisuke Momma, Yuki Matsui, Eiji Kondo, Norimasa Iwasaki

**Affiliations:** ^1^ University of Toronto Orthopaedic Sports Medicine, Women's College Hospital Toronto Ontario Canada; ^2^ Upper Extremity Center of Joint Replacement and Endoscopic Surgery, Hokushin Orthopaedic Hospital Sapporo Japan; ^3^ Department of Orthopaedic Surgery, Faculty of Medicine and Graduate School of Medicine Hokkaido University Sapporo Japan; ^4^ Center for Sports Medicine, Hokkaido University Hospital Sapporo Japan

**Keywords:** 4DCT, glenohumeral joint, Glenoid track, Hill‐Sachs lesion, shoulder instability

## Abstract

**Purpose:**

The concept of the glenoid track was proposed to evaluate the risk of shoulder dislocation with bipolar lesions, but dynamic in vivo evaluation during active motion has remained limited. The purpose of this pilot study was to evaluate glenohumeral (GH) contact area, contact centroid translation, and in vivo glenoid track visualisation during shoulder external and internal rotation using four‐dimensional computed tomography (4DCT).

**Methods:**

We obtained 4DCT data from the affected and intact shoulders of seven patients (mean age, 27.4 ± 5.5 years) with anterior shoulder instability and a Hill‐Sachs lesion during active cocking motion. The GH contact area (defined as the region with a glenohumeral joint space ≤4 mm) and translation of its centre were calculated from 3D bone models using customised software and compared between affected and intact shoulders at maximal internal rotation (MIR) and maximal external rotation (MER).

**Results:**

Affected shoulders showed a significantly smaller mean contact area at MER (429.5 ± 39.7 mm^2^) than at MIR (588.7 ± 42.1 mm^2^; *p* < 0.001). The centre of the contact area translated significantly anteriorly from MIR to MER in affected shoulders (1.56 ± 1.44 mm), but not in intact shoulders (–0.41 ± 0.75 mm; *p* < 0.001). In intact shoulders at MER, the in vivo glenoid track width corresponded to 74.6 ± 3.9% of the glenoid width, suggesting that functional glenohumeral contact behaviour during active motion may differ from conventional static estimates.

**Conclusion:**

This pilot feasibility study demonstrated that 4DCT can visualise the in vivo glenoid track and quantify dynamic glenohumeral contact mechanics during active motion. In affected shoulders, contact area decreased at MER and the centre of the contact area translated anteriorly during external rotation.

**Clinical Relevance:**

Dynamic assessment of the glenoid track using 4DCT enables in vivo visualisation of GH contact mechanics in patients with bipolar lesions. This method can identify anterior translation and reduced contact area during shoulder external rotation, which may provide complementary biomechanical information beyond conventional static assessment.

**Level of Evidence:**

Level IV, case series with intra‐patient comparison.

**Abbreviations**
3D: 3‐dimensional
4DCT: 4‐dimensional computed tomography
CT: computed tomography
GH: glenohumeral
MER: maximal external rotation
MID: midpoint between MIR and MER
MIR: maximal internal rotation
MRI: magnetic resonance imaging

## INTRODUCTION

Anterior shoulder dislocation can lead to recurrent anterior shoulder instability, especially in young and active patients [1]. In recurrent cases, bone defects of the glenoid and the humeral head are common, and combined glenoid and humeral head bone defects, defined as ‘bipolar lesions', have been recognised to increase the risk of recurrent instability after arthroscopic Bankart repair alone [11, 14]. Therefore, an assessment method that considers the interaction between these lesions in functional shoulder positions is important. The contact areas of the glenohumeral (GH) joint at abduction, external rotation, and horizontal extension are defined by Yamamoto et al. as the ‘glenoid track’ [20]. In recent years, the importance of evaluating bipolar lesions using glenoid track theory has been highlighted for anterior shoulder instability [5, 6, 17, 21, 22].

Although prior studies have evaluated the glenoid track using cadaveric investigations, static 3‐dimensional (3D) computed tomography (CT) [20], magnetic resonance imaging (MRI) [10, 16] and clinical scores [22], dynamic in vivo evaluation of glenohumeral contact behaviour during active motion has remained limited. Shoulder stability is significantly related to the soft tissue balance, including muscle strength; [9, 18] therefore, a dynamic in vivo assessment that inherently incorporates soft‐tissue effects may provide complementary information to static measurements. In addition, management of glenoid bone loss remains heterogeneous, particularly in the subcritical range, where surgical indications continue to be debated [2, 15]. In this context, a dynamic assessment of glenohumeral contact mechanics may help improve the biomechanical understanding of instability‐related contact behaviour in functionally relevant positions. Recently, Momma et al. [13] reported on the validity of evaluating the contact area of the GH joint using 4‐dimensional CT (4DCT). This modality can evaluate joint contact areas in dynamic situations while taking soft‐tissue balance into consideration, and has been used for clinical evaluation in various fields with a low radiation dose [12, 13].

The purpose of this study was to evaluate the contact area of the GH joint and assess the glenoid track in vivo during dynamic motion in patients with anterior shoulder instability and a Hill‐Sachs lesion using 4DCT. We hypothesised that the glenoid track can be assessed dynamically using in vivo 4DCT images and that patients with Hill‐Sachs lesions would have a narrower glenoid track in maximal external rotation (MER) of the GH joint than in maximal internal rotation (MIR) due to the bipolar lesion.

## MATERIALS AND METHODS

### Subjects

We obtained 4DCT images of the affected and intact shoulders of seven male patients with anterior shoulder instability with a history of anterior dislocation and a Hill‐Sachs lesion. Patients with shoulder osteoarthritis, patients over 40 years old, and patients with subluxation alone were excluded.

Ethics approval was obtained from the Research Ethics Review Committee of Hokkaido University Hospital (approval ID: 024‐0335). Written informed consent was obtained from all participants before imaging. Three patients subsequently underwent arthroscopic Bankart repair after imaging (ABR only, no bone grafting). No postoperative recurrences were reported at the time of the most recent clinical follow‐up documented in the medical record.

### 4D‐CT image data acquisition

**Figure 1:**
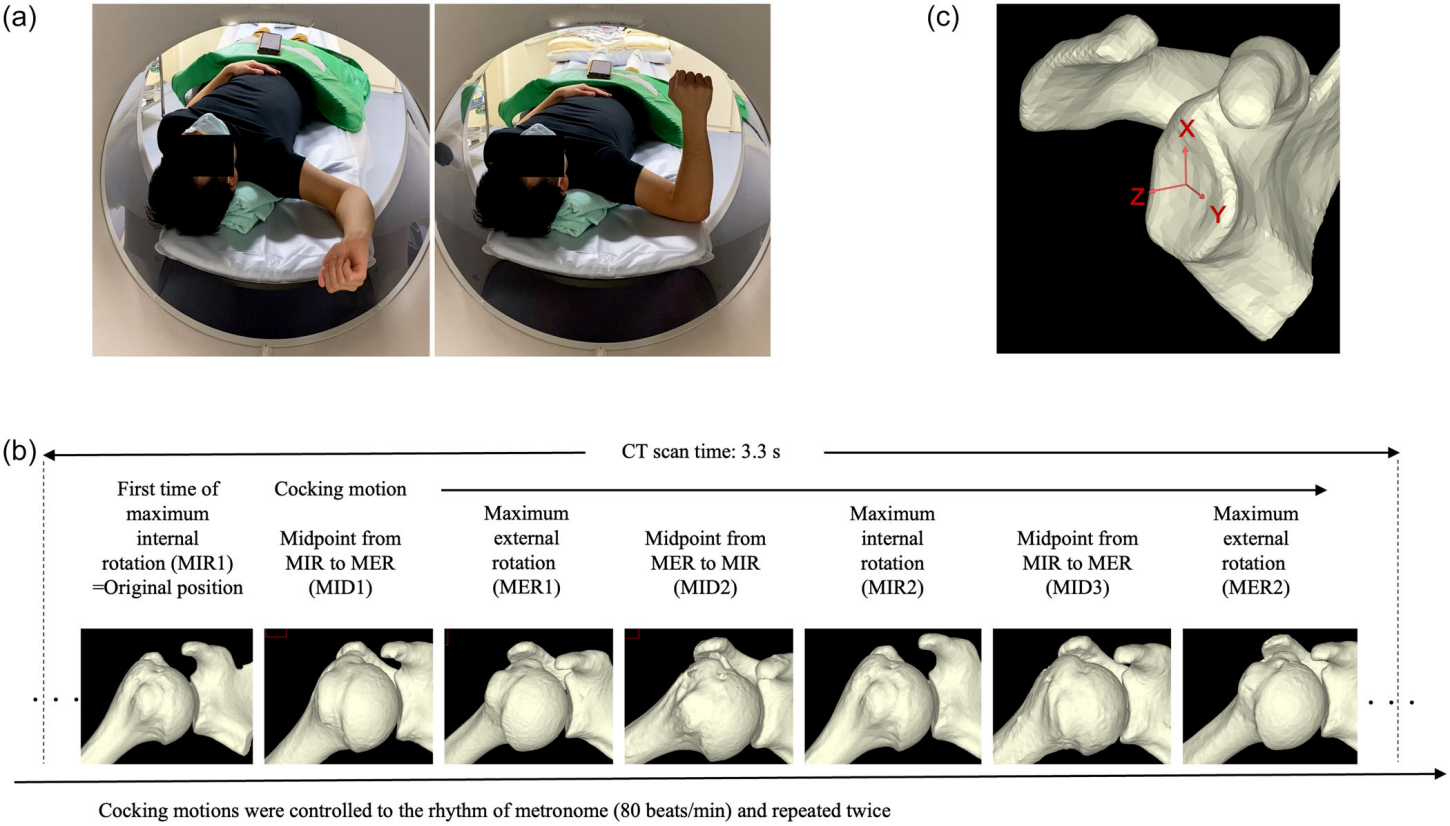
Results from 4DCT of the shoulder joint during internal and external rotation. (a) Acquisition of 4DCT. (b) Definition of each shoulder position through a scan time of 3.3 s for two cocking motions controlled to the rhythm of a metronome. (c) Coordinate system for the direction of translation of the joint contact area centroid. Translation along the *X*‐axis, *Y*‐axis and *Z*‐axis is indicated as the superior (+)/inferior (−) direction, anterior (+)/posterior (−) direction, and lateral (+)/medial (−) direction, respectively. 4DCT, 4‐dimensional computed tomography; MER, maximum external rotation; MID, midpoint from MER to MIR/MIR to MER; MIR, maximum internal rotation.

All imaging data used in this study were acquired from living patients during active shoulder motion using 4DCT; the subsequent 3D reconstruction and registration procedures were performed for computational analysis of these in vivo image datasets (Figure 1). All images were taken with a 320‐slice multidetector 4DCT scanner (Aquilion One; Toshiba Medical Systems) with a wide field of view and a slice thickness of 0.5 mm. Acquisition of shoulder 4DCT images followed the methods outlined by Momma et al. [13]. The shoulder was positioned in 90° of abduction and horizontal extension with a pillow under the scapula, and the elbow joint was positioned in 90° of flexion (Figure 1a). Scan parameters were set as follows: dynamic volume scan; wide field of view (size, LL; detector width, 16 cm); 80 kV; 100 mA; gantry speed, 0.275 s/rotation; effective mAs, 27; reconstruction function, FC01; reconstruction rate, 0.1 s (31 volumes); adaptive iterative dose reduction; slice thickness, 0.5 mm; and slice interval, 0.5 mm. Scan time was 3.3 s for two cocking motions controlled to a rhythm of 80 beats/min using a metronome. Each shoulder position was defined from a 3D bone model (Figure 1b). Total radiation exposure was set to not exceed 2.4 mSv. From the CT images, we measured glenoid width and length bilaterally. We then quantified the width of the glenoid bony defect on the affected side relative to the contralateral intact glenoid, and measured the humeral Hill–Sachs interval.

### Analysis of dynamic motion of the GH joint

Analytical methods also followed those described in the study by Momma et al. [13]. CT data for each shoulder were imported in DICOM format and segmented using segmentation software (Mimics 21R; Materialise). The 3D digital bone models of the scapula and humerus were created using a 3D‐3D registration technique at each position and exported as pointcloud and polygon models. Bone thresholds were determined by referencing previous studies of 3D bone models [24]. The 3D scapula and humerus bone models were analysed using customised software created in Microsoft Visual C++ with Microsoft Foundation Class programming environment (Microsoft) for further analysis [7, 8].

Shoulder rotation was analysed across frames covering two active cocking cycles. For standardised comparisons, we analysed three positions: MIR, MER, and the midpoint between MIR and MER (MID) position. MIR and MER were defined as the end‐range frames of internal and external rotation, respectively (the first frame of MER (MER1) and the second frame of MER (MER2); the first frame of MIR (MIR1) and the second frame of MIR (MIR2)). MID was defined as the midpoint frame between MIR and MER within each cycle (MID1 and MID3) and between MER and MIR in the return phase (MID2), as illustrated in Figure 1b.

**Figure 2:**
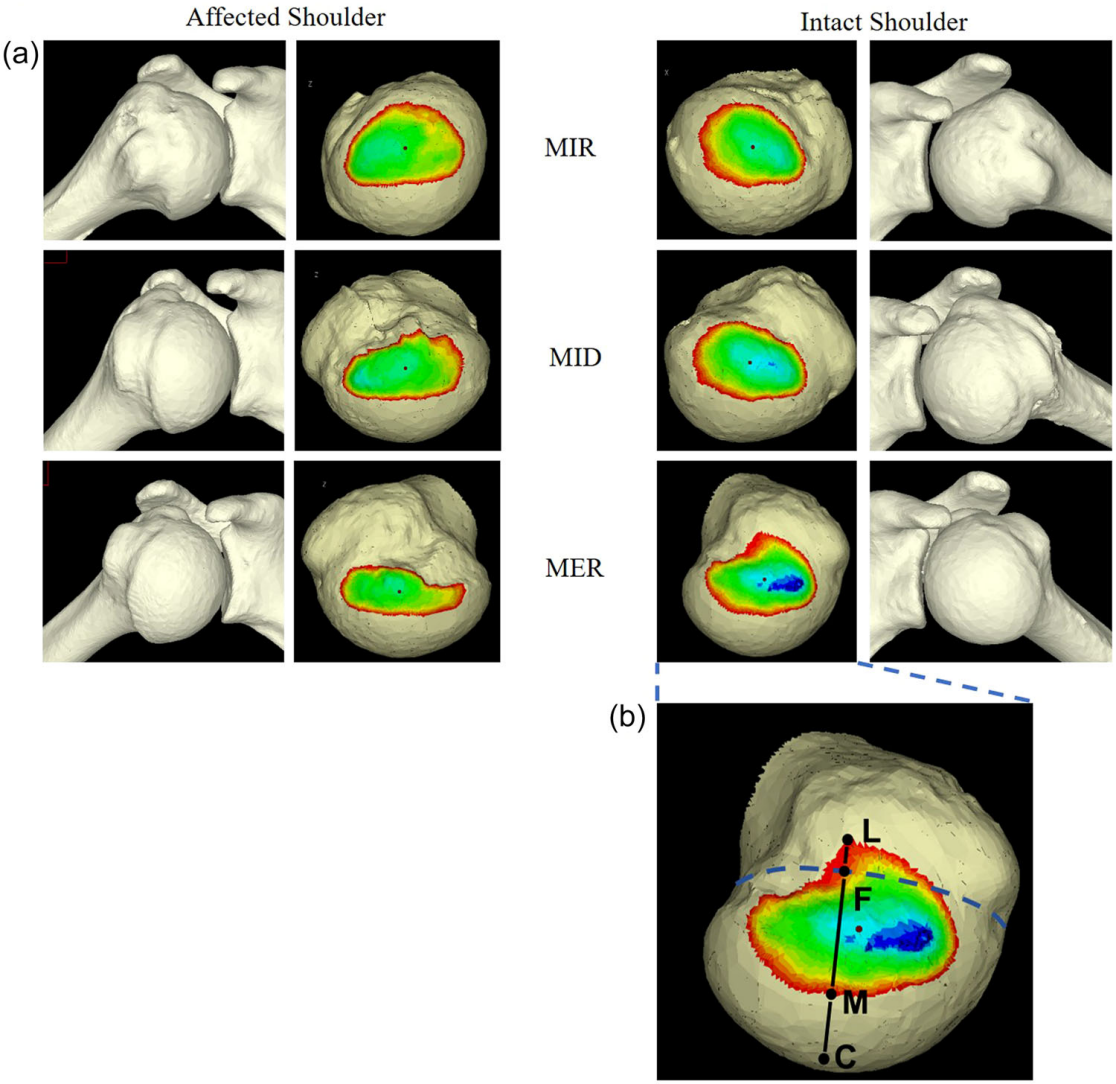
(a) Illustration of contact areas on the humeral head as extracted by digital software in a representative patient. The contact area is clearly narrower at MER than at MIR in the affected shoulder. (b) Landmark definition for measuring the in vivo glenoid track width on 4DCT in the intact shoulder at MER. Point C is defined as the centre of the articular surface of the humeral head. Point M is determined on the medial margin of the contact area, such that the distance from Point C to Point M is minimised. Point F is defined as the medial‐most point on the footprint and Point L is defined as the lateral‐most point on the contact area on Line CM. MER, maximum external rotation; MID, midpoint between MIR and MER; MIR, maximum internal rotation.

**Figure 3:**
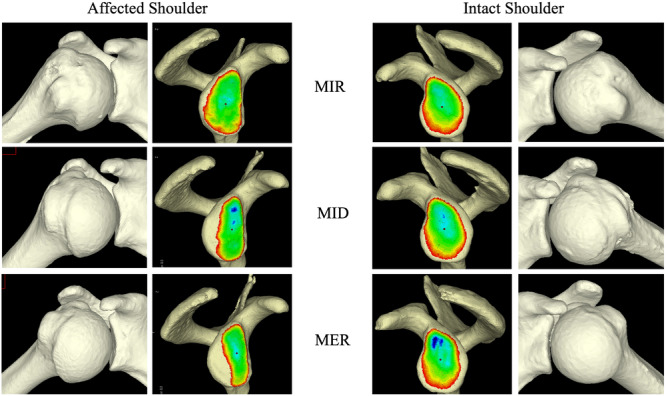
Illustration of contact area on the glenoid as extracted by digital software in a representative patient. The contact area at MER is clearly narrower and occupies a more anterior part of the glenoid surface compared to that at MIR in the affected shoulder. MER, maximum external rotation; MID, midpoint between MIR and MER; MIR, maximum internal rotation.

The contact area between the humeral head and the glenoid of both affected and intact shoulders was measured at each position. The contact area of the GH joint was defined as the region where the shoulder joint space was ≤4 mm, consistent with prior CT‐based contact analyses [3, 13] (Figures 2 and 3). This threshold was also considered a reasonable operational definition of the cartilaginous contact interface because published cartilage thickness data indicate that the combined thickness of the humeral head and glenoid cartilage approximates 4 mm [4].

To measure the width of the in vivo glenoid track on 4DCT, we defined the landmarks for measuring the glenoid track in the intact shoulder at MER based on a previous study [16, 20] (Figure 2b). First, the articular centre of the humeral head (Point C) was defined as the intersection between a superior‐inferior line connecting the 12 o'clock and 6 o'clock positions and a lateral‐medial line connecting the 3 o'clock and 9 o'clock positions of the articular surface of the humeral head. The most medial point (Point M) on the medial margin of the contact area was then determined such that the distance from Point C to Point M would be minimised. Finally, the distance from Point M to the medial margin of the footprint (Point F) on Line CM and the distance from Point M to the most lateral margin of the contact area (Point L) on Line CM were measured. All measurements were performed by a single investigator. We considered the width of the glenoid track as the distance from Point M to Point F on Line CM.

A validated 3D‐3D registration method was used to evaluate translation of the centre of the GH contact area and a transformation matrix from the original position to the rotated position was obtained as described by Momma et al. [13] (Figure 1c). The original position was defined as MIR1 (Figure 1b). We used the International Society of Biomechanics standard as the anatomical coordinate system for the shoulder [19].

Potential sources of measurement error include segmentation boundary selection and 3D‐3D registration error. Because all measurements were performed by a single investigator and formal intra‐ and inter‐observer reliability were not assessed, reproducibility was not quantified in this cohort. However, the 3D‐3D registration approach and contact‐area methodology have been validated in prior studies, including the work by Momma et al. [13], and the imaging voxel resolution (0.5 mm) provides a practical lower bound for geometric measurement precision.

### Statistical analysis

We compared the GH contact area and translation of the centre of the GH contact area between affected and intact shoulders using the paired *t*‐test. One‐way repeated‐measures analysis of variance was used to investigate per‐frame changes in contact area and centroid translation, followed by Tukey's honestly significant difference test for post‐hoc analysis. Values of *p* < 0.05 were considered significant. Data are presented as the mean ± standard deviation (SD) and corresponding 95% confidence intervals. Because this was an exploratory pilot feasibility study using a technically demanding dynamic imaging protocol, no a priori power analysis was performed. The present study was designed primarily to assess feasibility and characterise dynamic in vivo contact behaviour rather than to establish definitive clinical thresholds.

## RESULTS

### Participant demographics

Images from 4DCT were obtained from seven male patients (age, 27.4 ± 5.5 years; range, 20–38 years) diagnosed with anterior shoulder instability and a Hill–Sachs lesion. Glenoid width was significantly smaller in affected shoulders (26.6 ± 2.3 mm) than in intact shoulders (30.3 ± 2.3 mm; *p* = 0.010), and Hill–Sachs interval was 21.4 ± 1.9 mm. No significant difference in glenoid length was seen between the affected side (39.6 ± 2.1 mm) and the intact side (39.0 ± 1.1 mm; *p* = 0.510).

### GH joint contact area

At MIR, no significant difference in contact area was seen between the affected side (588.7 ± 42.1 mm^2^) and the intact side (595.7 ± 37.4 mm^2^; *p* = 0.644). However, at MER, the contact area was significantly smaller on the affected side (429.5 ± 39.7 mm^2^) than on the intact side (570.0 ± 45.9 mm^2^; *p* < 0.001).

**Figure 4:**
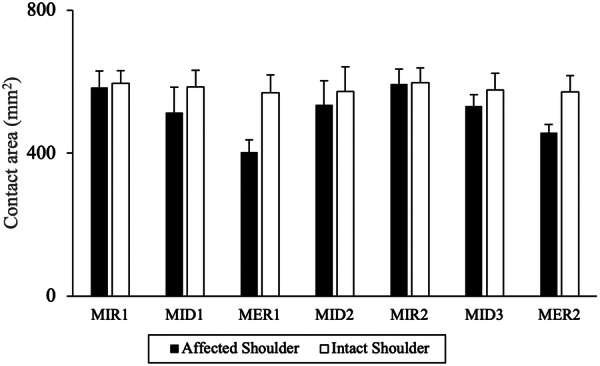
Mean and SD of the contact area at each point of MIR1, MID1, MER1, MID2, MIR2, MID3 and MER2 in both affected and intact shoulders. MER, maximum external rotation; MID, midpoint between MIR and MER; MIR, maximum internal rotation.

Frame‐by‐frame analysis showed that, on the affected side, mean contact area was significantly smaller in the MER phase (MER1, 402.6 ± 34.1 mm^2^; MER2, 456.4 ± 23.8 mm^2^) than in the MIR phase (MIR1, 583.9 ± 45.5 mm^2^; MIR2, 593.5 ± 41.3 mm^2^). Mean contact area was significantly smaller at MER (429.5 ± 39.7 mm^2^) than at MIR (588.7 ± 42.1 mm^2^; *p* < 0.001). In addition, the shape of the contact area was clearly narrower during MER than during MIR. Conversely, in intact shoulders, no significant difference in contact area or shape was seen between MER and MIR (Figures 2, 3, 4).

In the intact shoulder at MER, the in vivo glenoid track width was 22.5 ± 1.6 mm, corresponding to 74.6 ± 3.9% of the glenoid width (Table S1).

### Centre of the GH contact area

**Figure 5:**
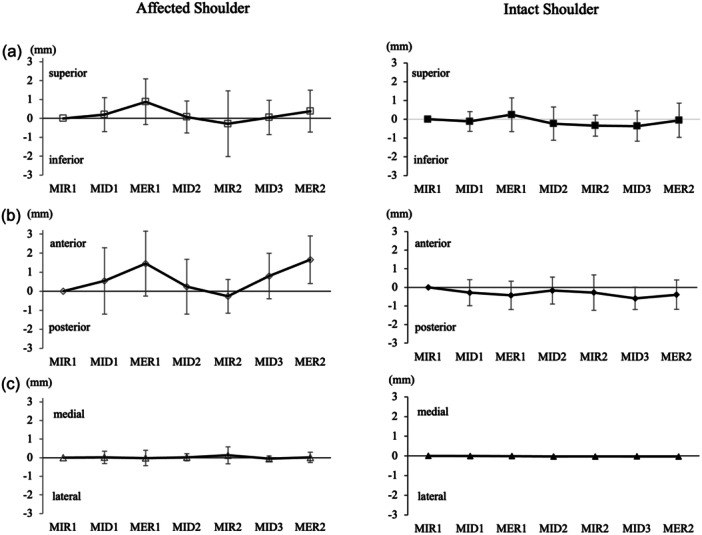
Mean and SD of translation of the centre of the contact area in each of three axes (a: superior/inferior; b: anterior/posterior; c: medial/lateral) at each point in both affected and intact shoulders: MIR1, MID1, MER1, MID2, MIR2, MID3 and MER2. MER, maximum external rotation; MID, midpoint between MER and MIR; MIR, maximum internal rotation.

To analyse the direction of translation of the centre of the GH contact area, translation was decomposed into superior, anterior, and medial directions (Figure 5). In the anterior‐posterior directions, significant differences were evident between affected and intact shoulders. In intact shoulders, anterior‐posterior translation remained small (near zero) with slight posterior translation at MER. In affected shoulders, the centre of the GH contact area translated anteriorly from MIR to MER. Mean anterior translation of the centre of the GH contact area from MIR to MER was 1.56 ± 1.44 mm in affected shoulders and −0.41 ± 0.75 mm in intact shoulders (*p* < 0.001) (Figure 5b). Translation in the superior‐inferior directions remained small in intact shoulders but tended to translate superiorly at MER in affected shoulders (Figure 5a). Translation in the medial‐lateral directions was minimal on both sides (Figure 5c).

## DISCUSSION

Principal findings of this study were: (1) the GH contact area was significantly smaller at MER than at MIR in affected shoulders with a Hill–Sachs lesion; (2) the centre of the contact area translated anteriorly from MIR to MER in affected shoulders, whereas intact shoulders showed minimal anterior‐posterior translation; and (3) the in vivo glenoid track width measured dynamically at MER in intact shoulders corresponded to approximately three‐quarters of the glenoid width.

To our knowledge, few studies have evaluated glenohumeral contact mechanics dynamically in vivo using 4DCT in patients with anterior shoulder instability and bipolar lesions during active cocking motion. The present study therefore adds pilot feasibility data regarding dynamic in vivo contact area assessment and glenoid track visualisation under functionally relevant conditions. Prior work has established the glenoid track concept using cadaveric models [16] and static imaging [20]. However, dynamic motion incorporates muscle activity and soft‐tissue balance, which are known to influence GH translation and stability [9]. The present 4DCT approach enables assessment of contact mechanics under these in vivo conditions.

Using 4DCT during active cocking at 90° abduction, we found that affected shoulders with bipolar lesions showed contact area narrowing at MER compared with MIR and an anterior shift of the centre of the contact area. These findings suggest that functional glenohumeral contact may be reduced and shifted anteriorly in a clinically relevant abducted and externally rotated position. Such contact mechanics are likely influenced by both osseous defects and soft‐tissue balance. Anterior glenoid bone loss may reduce the effective glenoid contact surface and anterior containment, while capsulolabral insufficiency, capsular laxity, and task‐dependent muscle forces may influence humeral head centreing and anterior translation at MER. Because our measurements were obtained in vivo during active motion, they reflect the combined effects of these factors. However, we did not directly quantify soft‐tissue status, including capsular laxity and labral integrity, or muscle activation. Dynamic 4DCT measures may add useful information beyond conventional static assessments by showing functional contact at MER.

The centre of the GH contact area translated anteriorly at MER in affected shoulders. Importantly, this anterior translation was observed in shoulders that also exhibited reduced glenoid width due to anterior bony loss. This finding provides an in vivo dynamic evaluation that is consistent with the clinically relevant abducted and externally rotated position in anterior shoulder instability. In contrast, translation of the centre of the contact area on intact shoulders showed a similar trend to that reported by Momma et al. [12], supporting the construct validity of our 4DCT‐based contact area analysis. Dynamic in vivo quantification of anterior translation may provide complementary biomechanical information for future outcome‐based studies, although its direct clinical applicability remains to be established.

The glenoid track was originally reported as approximately 84% of the glenoid width in a cadaveric model [20], and Omori et al. reported approximately 83% using static open MRI in vivo [16]. However, these measurements were obtained in a series of static poses. In contrast, our dynamic in vivo measurement at MER showed a glenoid track width corresponding to 74.6 ± 3.9% of glenoid width. Rather than redefining the conventional static glenoid track value, this finding suggests that the functional glenoid track may vary under dynamic in vivo conditions. This difference may reflect task‐dependent muscle activation patterns and dynamic joint loading during active motion, which are not captured in static imaging, although these factors were not directly measured in the present study. Importantly, concepts such as peripheral‐track lesions [22] and the Hill‐Sachs interval/glenoid track ratio [23] suggest that recurrence risk may increase even when a lesion is classified as on‐track. Considering these points, our findings suggest that dynamic in vivo measurement at MER may provide complementary information regarding functional contact mechanics beyond conventional static estimates.

Several limitations should be considered. First, the sample size was small and all participants were male, limiting generalisability. This sample size limitation is due to the technical complexity of the imaging protocol and radiation exposure considerations. However, the primary aim of the present study was to demonstrate the feasibility and potential value of dynamic in vivo evaluation of the glenoid track, rather than to establish definitive clinical thresholds. Therefore, we believe that the current sample size is appropriate for a pilot or feasibility study. A further large study is necessary for the clinical implications of this technique. Second, clinical outcomes and range of motion were not evaluated. Therefore, the relationship between these dynamic imaging findings and clinical outcomes remains unclear. Third, we did not quantify measurement reproducibility (intra‐ or inter‐observer reliability), and potential error related to segmentation and registration remains. Fourth, the ≤4 mm threshold used to define contact area should be interpreted as an operational imaging definition rather than a direct physiological measure of true cartilage contact. Finally, only a single motion task (active cocking in 90° abduction) was evaluated; contact mechanics may differ in other positions. Despite these limitations, affected shoulders demonstrated a consistent pattern of reduced contact area at MER and anterior centroid translation, suggesting that the methodology can detect characteristic dynamic contact changes associated with bipolar lesions.

## CONCLUSION

In vivo glenoid track visualisation was feasible using 4DCT and customised software. In affected shoulders with a Hill–Sachs lesion, the GH contact area was significantly smaller at MER than at MIR, and the centre of the contact area translated anteriorly during external rotation. These findings suggest that in vivo 4DCT evaluation of the glenoid track and contact mechanics may be useful in future studies assessing the glenoid track dynamically.

## AUTHOR CONTRIBUTIONS

All authors have contributed substantially to the conception, design, analysis and interpretation of the data in this manuscript and will take responsibility for the content.

## FUNDING

The authors have no funding to report.

## CONFLICT OF INTEREST STATEMENT

The authors declare no conflicts of interest.

## ETHICS STATEMENT

This study was approved by the Research Ethics Review Committee of Hokkaido University Hospital (Approval ID: 024‐0335). Written informed consent was obtained from all participants. Informed consent for the use of samples in this research was obtained from all participants. Informed consent for publication of identifying images in an online open‐access publication was also obtained.

## Supporting information

Supplementary Table S1. In vivo glenoid track width and related ratios in the intact shoulder at maximal external rotation.

## Data Availability

The datasets generated and analysed during the current study are available from the corresponding author upon reasonable request.
