# Supplementary material for: Four‐dimensional computed tomography can visualise the in vivo glenoid track and quantify dynamic glenohumeral contact mechanics during active motion in subjects with shoulder instability: A preliminary study
Source: J Exp Orthop. 2026 Jun 16;13(2):e70807. doi: 10.1002/jeo2.70807 (PMC13270351; doi:10.1002/jeo2.70807)
Supplement: Supplementary file 1 — Supplementary Table S1. In vivo glenoid track width and related ratios in the intact shoulder at maximal external rotation. [file JEO2-13-e70807-s001.docx]

Supplementary Table S1. In vivo glenoid track width and related ratios in the intact shoulder at maximal external rotation

| Case | Width of the glenoid track, mm | Distance from Point M to Point L, mm | Ratio of the width of the glenoid track to the distance from Point M to Point L, % | Ratio of the width of the glenoid track to glenoid width, % |
| --- | --- | --- | --- | --- |
|  |  |  |  |  |
| 1 | 20.5 | 23.0 | 89.1 | 70.8 |
| 2 | 21.5 | 23.9 | 90.0 | 75.3 |
| 3 | 25.2 | 28.1 | 90.0 | 77.9 |
| 4 | 24.2 | 27.5 | 88.0 | 71.5 |
| 5 | 22.5 | 26.1 | 86.2 | 72.3 |
| 6 | 21.5 | 25.1 | 85.7 | 72.5 |
| 7 | 22.3 | 24.5 | 91.0 | 81.4 |
| Mean ± SD | 22.5 ± 1.6 | 25.5 ± 1.8 | 88.5 ± 2.0 | 74.6 ± 3.9 |
